# Success of torsional correction surgery after failed surgeries for patellofemoral pain and instability

**DOI:** 10.1007/s11751-013-0181-8

**Published:** 2013-12-15

**Authors:** Peter M. Stevens, Jeremy M. Gililland, Lucas A. Anderson, Jennifer B. Mickelson, Jenifer Nielson, Joshua W. Klatt

**Affiliations:** Department of Orthopaedic, Primary Children’s Hospital, University of Utah School of Medicine, 100 Mario Capecchi Drive Suite 4550, Salt Lake City, UT 84113 USA

**Keywords:** Pan genu torsion, Miserable malalignment, Tibial torsion, Femoral anteversion, Osteotomy

## Abstract

Torsional deformities of the femur and/or tibia often go unrecognized in adolescents and adults who present with anterior knee pain, and patellar maltracking or instability. While open and arthroscopic surgical techniques have evolved to address these problems, unrecognized torsion may compromise the outcomes of these procedures. We collected a group of 16 consecutive patients (23 knees), with mean age of 17, who had undergone knee surgery before torsion was recognized and subsequently treated by means of rotational osteotomy of the tibia and/or femur. By follow-up questionnaire, we sought to determine the role of rotational correction at mean 59-month follow-up. We reasoned that, by correcting torsional alignment, we might be able to optimize long-term outcomes and avert repeated knee surgery. Knee pain was significantly improved after torsional treatment (mean 8.6 pre-op vs. 3.3 post-op, *p* < 0.001), while 70 % of patients did have some continued knee pain postoperatively. Only 43 % of patients had continued patellar instability, and 57 % could trust their knee after surgery. Activity level remained the same or increased in 78 % of patients after torsional treatment. Excluding planned rod removal, subsequent knee surgery for continued anterior knee pain was undertaken on only 3 knees in 2 patients. We believe that malrotation of the lower limb not only raises the propensity for anterior knee symptoms, but is also a under-recognized etiology in the failure of surgeries for anterior knee pain and patellar instability. Addressing rotational abnormalities in the index surgery yields better clinical outcomes than osteotomies performed after other prior knee surgeries.

## Introduction

Anterior knee pain and patellofemoral instability are common causes of knee problems in both adolescents and adults. Theories as to the etiology of the above have included quadriceps muscle imbalance, retinacular/soft-tissue tightness, elevated “Q-angles,” pathomorphology of the trochlea, and abnormal extensor complex (patella alta/baja) [1–3]. However, torsional deformities of the femur and/or tibia often go unrecognized in both adolescents and adults who present with anterior knee pain, and patellar maltracking and/or instability. While arthroscopic [4] and open [5, 6] surgical techniques have evolved to address the latter problems, the results may be compromised by underlying torsion. Failure to recognize and address the transverse plane deformities may prove disappointing to the patient and surgeon alike.

We hypothesized that there would be a satisfactory clinical improvement after rotational osteotomies of the tibia and/or femur in knees with unrecognized torsional abnormalities and ongoing knee problems after prior knee surgery for anterior knee pain or patellofemoral instability.

## Materials and methods

**Fig. 1:**
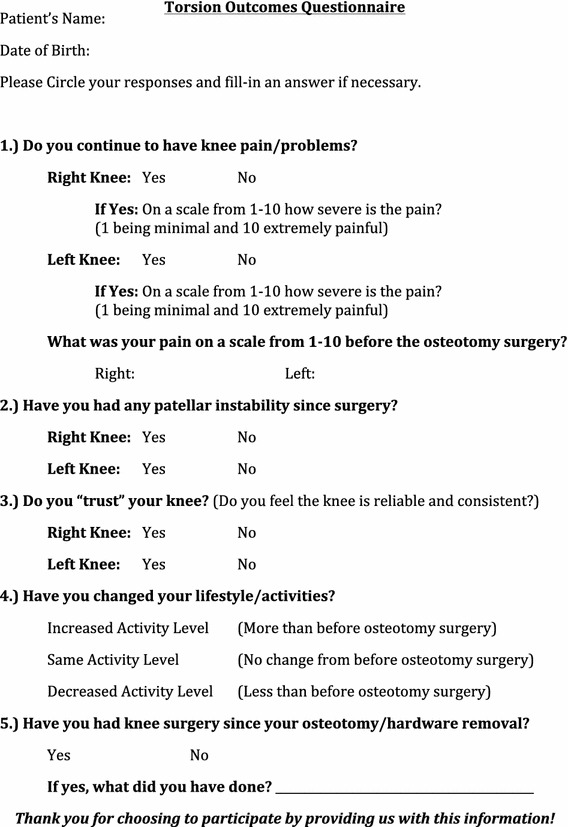
Torsion outcomes questionnaire

This study was a retrospective review on a group of 18 patients who all had developed anterior knee pain and/or instability before skeletal maturity and had all undergone one or more prior knee surgeries before presenting to our clinic where torsional abnormalities were recognized (Fig. 1). Torsional deformities were treated by a single surgeon in our institution (PMS) by means of rotational osteotomy of the tibia and/or femur between May 1998 and November 2010.

**Fig. 2:**
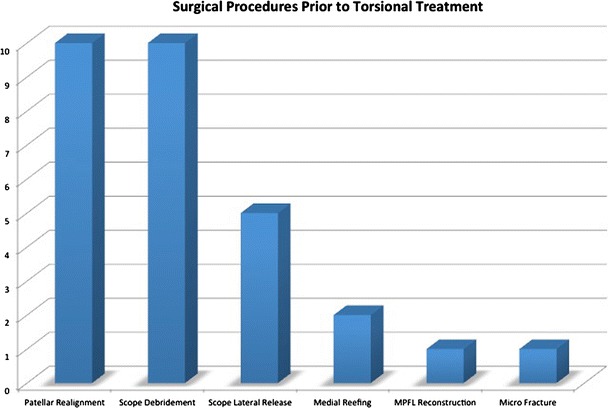
*Bar graph* illustrating types and number of prior failed surgeries before rotational malalignment addressed

**Fig. 3:**
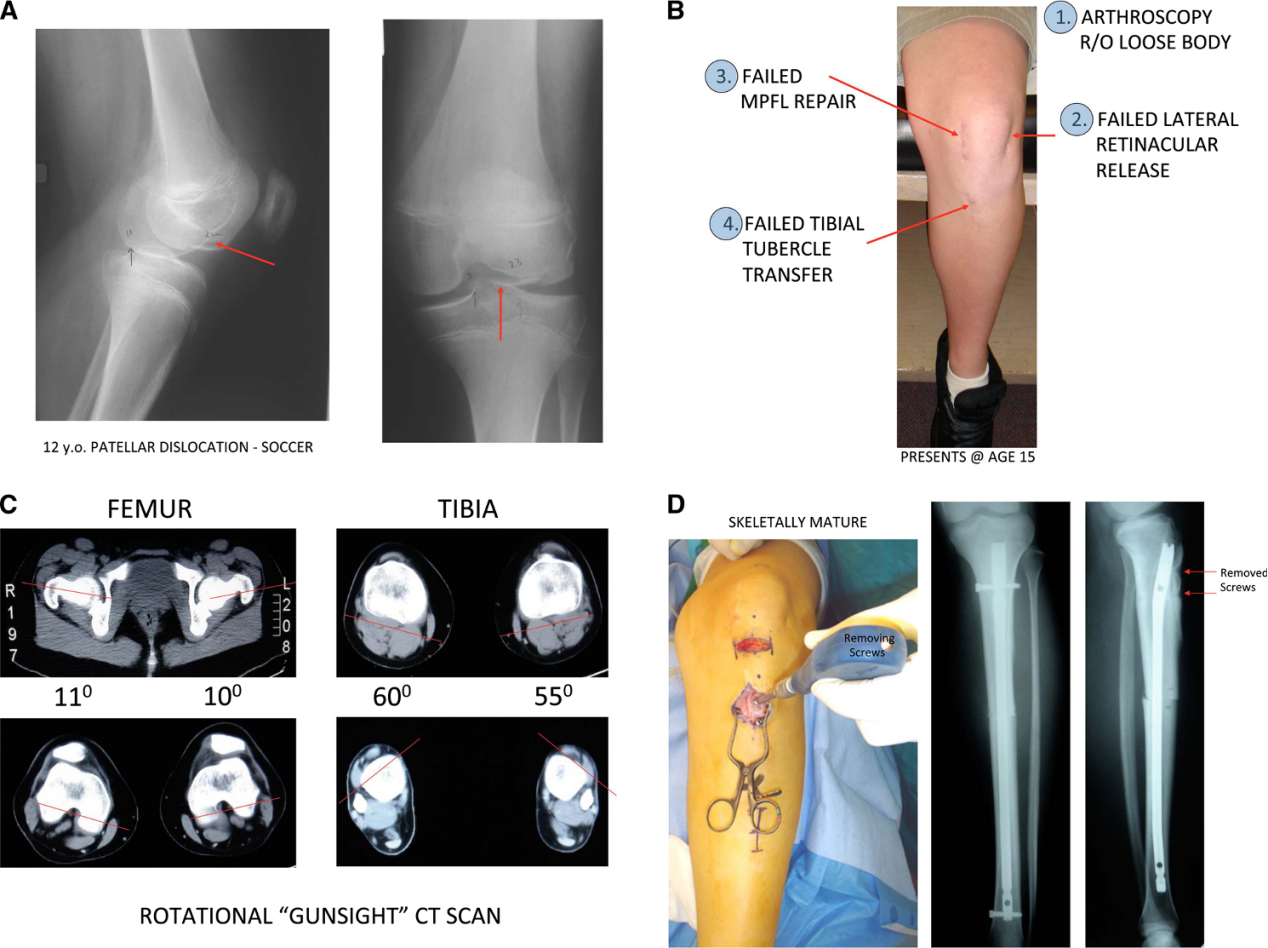
**a** A 12-year-old girl was “tackled” playing soccer and suffered a traumatic dislocation of her *left* patella. The *arrow* depicts the osteochondral fracture. **b** Three years (and four knee operations) later, she continues to have *left* patellar instability. She is now developing knee symptoms on the *right*. **c** Her torsional profile demonstrated 30° of excessive outward tibial torsion bilaterally. Her “gunsight” CT scan corroborates the excessive bilateral tibial outward torsion, in the presence of normal femoral version. **d** At the time of her 30° rotational tibial osteotomy, the tubercle screws were removed, leaving the transferred tubercle in situ. Two months later, she underwent a rotational osteotomy of her *right* tibia. At latest follow-up, she has stable patellae and is asymptomatic

We contacted these patients with a custom outcome questionnaire and 17 of 18 returned the questionnaire (Fig. 2). One of the 17 returned questionnaires contained incomplete data and was excluded. We therefore reviewed the records of 16 participating patients with 23 operated knees with mean 59-month follow-up (range 11–145 months). This group consisted of 13 females and 3 males. The mean age at the time of index rotational osteotomy was 17, with a range of 9–30 years of age. The average number of prior knee surgeries per patient was 2 with a range of 1–4 (Table 1). A patient case example is included in Fig. 3.

**Table 1:** Patient demographics and procedures

Patient	Side	Gender	Age^a^	Follow-up (months)	Prior knee surgeries	Femoral torsional correction (°)	Tibial torsional correction (°)	Lateral release at time of osteotomy	Complications	Subsequent surgeries
1	R	F	19	17	Tibial tubercle transfer	20	30	No	Nonunion	Exchange nailing for femoral nonunion
2	L	F	17	18	Arthroscopic debridement × 2	20	30	No		
3	R	M	9	113	Arthroscopic debridement	20	20	Yes		Distal femoral hemiepiphysiodesis, knee arthroscopy, MPFL reconstruction
3	L	M	9	110	Arthroscopic debridement	20	20	No		Distal femoral hemiepiphysiodesis
4	R	F	16	18	Tibial tubercle transfer, Arthroscopic debridement	20	30	Yes		
5	R	F	10	42	MPFL Reconstruction	30		No		Tibial tubercle transfer and lateral release
6	L	M	22	41	Tibial tubercle transfer, medial reefing	30	30	No		
7	L	F	24	61	Arthroscopic lateral release	30	40	No		
7	R	F	24	63	Arthroscopic lateral release	35	35	Yes		
8	R	F	15	117	Tibial tubercle transfer		25	No		
8	L	F	15	114	Tibial tubercle transfer		40	No		
9	R	F	17	36	Tibial tubercle transfer		30	Yes		
9	L	F	17	35	Tibial tubercle transfer		30	No		
10	R	F	17	86	Tibial tubercle transfer, Arthroscopic lateral release		30	No		
10	L	F	17	82	Arthroscopic lateral release		25	No		
11	R	F	16	14	Arthroscopic debridement		25	Yes		
11	L	F	16	13	Arthroscopic debridement		30	Yes		
12	L	F	18	26	Tibial tubercle transfer, Arthroscopic debridement, Medial reefing		25	No	Loose interlock with nerve irritation	Advancement left distal interlock screw, decompression peroneal nerve
12	R	F	18	11	Tibial tubercle transfer		25	No		
13	R	F	18	64	Tibial tubercle transfer		25	Yes		
14	L	F	30	145	Tibial tubercle transfer, Arthroscopic debridement, Micro fracture		25	No		Arthroscopic microfracture of patella
15	R	M	16	113	Arthroscopic debridement		30	Yes		
16	R	F	21	22	Arthroscopic debridement, Arthroscopic lateral release		30	No		

*MPFL* medial patellofemoral ligament

^a^Age is age at time of osteotomy in years

Prior to osteotomy, each patient had a “gunsight” rotational CT scan or MRI to document the degree of excessive torsion and determine whether to cut the femur, tibia, or both [2, 7, 8] (Fig. 3). Surgical interventions included femoral rotational osteotomy to correct anteversion, tibial rotational osteotomies to address outward tibial torsion, and simultaneous ipsilateral tibial and femoral osteotomies to address pan genu torsion (Tables 1 and 2). Surgical correction of the contralateral extremity was undertaken within 3 months in seven patients with bilateral symptoms.

**Table 2:** Operative details (*n* = 23 rotational surgeries)

Operative detail	Number^a^	Amount of correction^b^
Femoral osteotomy	9 (39 %)	25 (20–35)
Tibial osteotomy	22 (96 %)	29 (20–40)
Both femoral and tibial	8 (35 %)	54 (40–70)^c^
Lateral release	8 (35 %)	

^a^Data presented as mean with percentage of total cases in parentheses

^b^Data presented as mean with range in parentheses

^c^Data presented as mean total combined correction of tibial and femoral osteotomies with range in parentheses

The femoral osteotomies were performed percutaneously, at the mid-shaft level and stabilized with an antegrade, locked, intramedullary rod, using a trans-trochanteric (not piriformis) entry point [9]. In skeletally mature patients, all tibial osteotomies were mid-shaft, leaving the fibula intact, and secured with an intramedullary rod [10]. If the patellar retinaculum was tight, as judged by a negative patellar inversion angle, then a lateral retinacular release was undertaken at the same sitting. The intramedullary rods were removed approximately 1 year after the osteotomies.

For the patients who were skeletally immature, the open proximal physis precluded the safe use of an intramedullary rod in the tibia. Instead, a supra-malleolar osteotomy of the tibia was performed and stabilized with two smooth, crossed Steinman pins, leaving the fibula undisturbed for stability and support [11]. Following 4 weeks in a below-the-knee cast, the pins were removed in the clinic and the patients permitted weight bearing in a walking boot for an additional month.

Clinical outcomes were evaluated by a custom questionnaire (Fig. 1). This questionnaire asked about preoperative and postoperative pain levels, recurrent patellar instability, ability to “trust” the knee, postoperative activity levels, and any subsequent knee surgeries after the rotational osteotomy and hardware removal.

Data were analyzed by an independent statistician using commercially available software (STATA Version 11, College Station, TX). Student’s t test was used for comparing the continuous variables of preoperative and postoperative pain.

## Results

Knee pain on a scale of 1–10 was significantly improved after torsional treatment (mean 8.6 pre-op vs. 3.3 post-op, *p* < 0.001), while 70 % of patients did have some continued knee pain postoperatively (Table 3). Ten of 23 knees had continued patellar instability after torsional treatment, and 13 of 23 knees were “trusted” by the patient of after surgery. Activity level was improved after 15 of 23 of these cases, remained the same after in 3 knees, and was decreased after in 5 knees.

**Table 3:** Clinical outcome measures (*n* = 23 knees with mean follow-up of 59 months)

Pain	Preoperative	Postoperative	*p* value
Pain on scale of 1–10^a^	8.6 (7.9–9.4)	3.3 (2.0–4.6)	**<** ***0.001***

Outcomes questionnaire item	Postoperative response
Do you continue to have knee pain/problems?	70 % Yes
Have you had any patellar instability since surgery?	43 % Yes
Do you “trust” your knee?	57 % Yes
Have you changed your lifestyle/activities?	65 % Increased
	13 % Same
	22 % Decreased
Have you had knee surgery since your osteotomy/hardware removal?	22 % Yes

^a^Data presented as means with 95 % confidence intervals in parentheses

Our complication rate in this series was 8.7 % with 2 complications encountered. The first complication was a femoral nonunion treated successfully with exchange reamed nailing. The second complication was a peroneal nerve irritation by a loose proximal tibial interlocking screw. This was treated successfully with hardware removal and peroneal neurolysis. Excluding planned rod removal, subsequent knee surgery for continued anterior knee pain was undertaken on three knees in two patients. One patient underwent bilateral guided growth to address genu valgum, bilateral patellar realignment, and MPFL reconstruction. The second patient required a knee scope debridement and microfracture (Table 1).

## Discussion

**Fig. 4:**
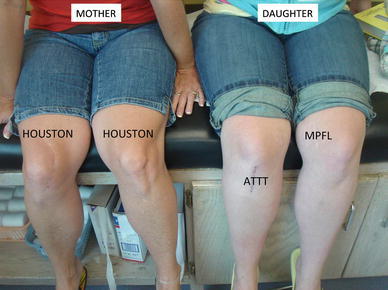
There is a prevalence of torsional anomalies (femoral anteversion and/or outward tibial torsion) in females, and this is often familial. This *mother* and *daughter*, both with excessive outward tibial torsion, have undergone a variety of patellar stabilization surgery, but remain symptomatic

Persistent torsional deformities of the femur and/or tibia often go unrecognized in patients with anterior knee pain and/or patellofemoral instability. As the associated acute and chronic symptoms are most often at the knee, there is a tendency for the orthopedic surgeon to focus on that level, without screening for rotational abnormalities of the femur and/or tibia. There is a lingering belief that the physiologic torsional deformities, observed during childhood, are uniformly benign and self-limiting [12]. This belief may result in a failure to appreciate the consequences of persistent and pathological torsion; even Staheli states that “persistent problems are often genetically determined and may only be corrected by osteotomy.” Unfortunately, failing to screen for torsional deformities can lead to the detriment of some reconstructive endeavors of the orthopedic sports medicine and adult reconstruction specialists [5, 13–15] (Fig. 4).

Femoral anteversion and/or outward tibial torsion have a dramatic effect upon the kinematics of the knee, most notably of which may be seen at the patellofemoral join [11]. Eckhoff stated that “the patella is a passive component of the extensor mechanism, where the static and dynamic relationships of the underlying tibia and femur determine the patellar tracking pattern” [2]. Turner and Smillie in their measurements of tibial torsion in over 800 patients found that patients with patellofemoral instability had a significant increase in outward tibial torsion [16]. Increased femoral anteversion, external tibial torsion, pan genu torsion, and knee version (external rotation of the tibia with respect to the femur) have all been shown to be associated with anterior knee pain in various studies. Therefore, while patellar instability and/or persistent patellofemoral pain may require prompt and sometimes aggressive management, the possibility that there is a proximate cause of the problem should be kept in mind [1, 2, 8, 16]. In fact, Flandry and Hughston [15] showed a high failure rate for extensor mechanism realignment when the underlying cause of patellofemoral pain was excessive femoral anteversion, external tibial torsion, or both.

A careful clinical assessment is most revealing and is paramount to successful clinical management. When the patient stands with their feet parallel, the patella should be facing forward. The “foot progression angle” should be neutral when walking [12, 17]. When presenting individually, femoral anteversion is manifest by in-toeing and outward tibial torsion by out-toeing. However, if there is concomitant femoral anteversion and outward tibial torsion, also known as pan genu torsion or “miserable malalignment” [7], the footprint will be neutral and this combined long bone deformity may be concealed to the unwary observer [7]. It is therefore important to have the patient appropriately unclad and note the knee progression angle as well [7, 10, 11].

Malrotation is readily documented by means of assessing the torsional profile in the prone position. Tibial torsion can be documented by measuring the thigh-foot axis (normal = neutral to 15° outward) [16]. Femoral anteversion may be recognized by identifying, in the prone position, how much inward rotation there is at each hip in extension, compared to outward rotation (normally equal inward and outward rotation ±30°) [5, 7]. The findings are not always symmetrical; anecdotally, it is often noted that the affected knee is the one that has more torsion. While plain radiographs do not accurately identify torsion, they are nevertheless helpful. A long-standing anteroposterior radiograph of the legs, with the patellae neutral, will identify any concurrent limb length discrepancy and/or angular deformities. The apparent increase in the femoral neck-shaft angle may be due to increased femoral anteversion. One can observe the presence (or closure) of the physes and measure the limb lengths and mechanical axis. A lateral view of each knee and Merchant [18] or Laurin views [19] of the patella may demonstrate patellar malorientation or fragmentation, perhaps as a consequence of long bone torsion.

Significant abnormalities documented on the clinical examination, correlated with the history, may be an indication for advanced imaging. The standard is the “gunsight” CT scan or MRI, employing accepted bony landmarks to define and quantify the degree of malrotation of the femur and/or tibia. While not mandatory, this imaging may help to refine the plans for rotational corrective osteotomies [2, 7–9, 11]. Lee et al showed through a cadaver knee study that a 20° inward rotational deformity of the femur resulted in increased lateral patellofemoral contact pressures, and a drastic increase in lateral patellofemoral contact pressure resulted when 30° or more inward rotational deformity was seen [20]. We therefore employ the femoral osteotomies for patients who have greater than 20° of inward rotational deformity indicated by the preoperative hip range of motion and CT scan. Turner studied rotational profiles in over 1,200 patients and showed consistently that patients with patellofemoral pain and instability had increased tibial outward torsion measuring an average of 24.5° [16]. We employ inward tibial osteotomies for patients who have greater than 20° of excess outward rotational deformity [11]. As can be seen in the results from this study, our typical corrective rotations are 25° in the femur and 30° in the tibia.

The level and specific technique/fixation of rotational osteotomies remain up to the discretion of the surgeon, which is typically a reflection of training and experience.

With the exception of the immature tibia, we believe that intramedullary fixation offers several advantages [10, 21]. The surgical exposure is minimal, and the comparatively strong load-sharing implants are well tolerated. Immediate mobilization of the joints without cast protection is also appealing to the patient and surgeon alike. In the setting of the skeletally immature tibia, our preferred method of derotation is a distal tibia osteotomy secured with crossing Kirschner wires. We feel that distal tibial osteotomies are simpler, well tolerated, and we have shown that they work well in terms of correcting knee alignment and frontal plane knee moments [11]. In 1994, two separate studies showed improved outcomes for tibial rotational osteotomies when the fibula was left intact [22, 23]. Fibular osteotomy is rarely used in our practice and is reserved for limbs where we cannot easily achieve the desired rotation.

In our experience, patients with anterior knee pain and underlying torsional abnormalities respond very well to derotational corrective osteotomies. In previous work, we evaluated a group of 14 patients with anterior knee pain in 27 limbs with underlying pan genu torsion. These patients all had excellent outcomes with derotational osteotomies of their femurs and tibias [7]. However, none of these patients had prior surgical interventions for their anterior knee pain. The excellent outcomes seen in our 2004 study provide a stark contrast to the results of the present study comprised entirely of patients who had prior surgeries for their anterior knee symptoms. About 70 % of the current study patients had some continued knee pain, 43 % of these knees had continued patellar instability, and activity level was decreased in 22 % of these patients.

The history of prior failed knee surgery is not a contraindication to corrective osteotomy. In our experience, if a lateral retinacular release has already been undertaken, this may not need to be repeated. If the patellar tendon has been transferred, it may be left in situ, removing the retained screws if an intramedullary device is to be utilized. If the patella remains unstable, revision of patellar alignment should be postponed pending the results of the rotational correction; this may be undertaken at the time of rod removal. Likewise, such draconian measures such as patellectomy or patellofemoral arthroplasty should be postponed unless or until the femoral and/or tibial malrotation have been corrected [14, 24].

There are several limitations to this study. The retrospective nature yields itself to several biases, including selection and recall bias. However, we did attempt to follow these patients prospectively through the use of our outcomes questionnaire. Our outcome measures are inherently limited in that we are not using a validated outcome measure such as the Lysholm knee score [25]. The follow-up on this study was also relatively short-term, limiting conclusions regarding prevention of arthritis, lasting symptom relief, and need for further interventions. A final limitation is that this is a relatively small and heterogeneous group of patients. While we have a prior study of pan genu torsion patients without prior surgery, we feel that this study of patients who had prior failed nontorsional correcting surgery is a useful addition to the literature and a caveat to the knee surgeon.

## Conclusion

In conclusion, we believe that malrotation of the lower limb not only raises the propensity for knee injuries and conditions, but is also a under-recognized etiology in the failure of surgeries for anterior knee pain and/or patellar instability. Further examination of our two series demonstrates that addressing rotational abnormalities in the index surgery yields better clinical outcomes than osteotomies performed after extensor complex transfers or soft-tissue procedures. There are advantages to considering rotational osteotomy early in patients with prominent rotational abnormalities.
